# Age‐related presence and genetic diversity of *Campylobacter* spp. in young and adult yellow‐legged gulls (*Larus michahellis*) in Croatia

**DOI:** 10.1111/1758-2229.70017

**Published:** 2024-10-07

**Authors:** Biljana Ječmenica, Sanja Duvnjak, Andrea Humski, Louie Thomas Taylor, Jelena Kralj, Fani Krstulović, Tajana Amšel Zelenika, Viktor Mašović, Luka Jurinović

**Affiliations:** ^1^ Poultry Centre Laboratory for Bacteriology, Croatian Veterinary Institute, Poultry Centre Zagreb Croatia; ^2^ Laboratory for Bacterial Zoonoses and Molecular Diagnostics of Bacterial Diseases, Department for Bacteriology and Parasitology Croatian Veterinary Institute Zagreb Croatia; ^3^ Laboratory for Food Microbiology, Department for Veterinary Public Health Croatian Veterinary Institute Zagreb Croatia; ^4^ Croatian Academy of Sciences and Arts, Institute of Ornithology Zagreb Croatia

## Abstract

The epidemiology of *Campylobacter* species in wild birds is still poorly understood. This study describes the occurrence and genetic diversity of *Campylobacter* in adult and nestlings of yellow‐legged gulls, highlighting differences between breeding locations. The gulls were captured in Croatia between 2021 and 2023. A cloacal swab was taken from each individual and tested for the presence of *Campylobacter*. Isolated *Campylobacter* species were genotyped using the multilocus sequence typing (MLST) method. A total of 1071 gulls were captured and sampled, of which 152 samples were identified as *Campylobacter* species, with *Campylobacter jejuni* (9.90%) being the most frequently isolated bacterium, followed by *Campylobacter lari* (3.36%) and *Campylobacter coli* (0.93%). Complete sequence type (ST) profiles were generated for 141 isolates: 100 *C. jejuni*, 33 *C. lari*, and 8 *C. coli*. A significant difference in the occurrence of positive *Campylobacter* species was found depending on the sampling sites, while both sampling site and age were significant for the occurrence of *C. jejuni*. Adults and nestlings showed high genetic diversity for *C. jejuni* and *C. lari*, and there were no significant differences between strains isolated from adults and nestlings or between sites, suggesting a high genotype flow in the studied gull population.

## Graphical abstract

Minimum spanning tree showing *Campylobacter jejuni* sequence types (STs) according to age group (A) and locations (B). Regarding genetic diversity, no significant differences were found between age groups and locations. There is no clustering of specific genotypes, suggesting that all sampled yellow‐legged gulls have similar exposure to *Campylobacter*.

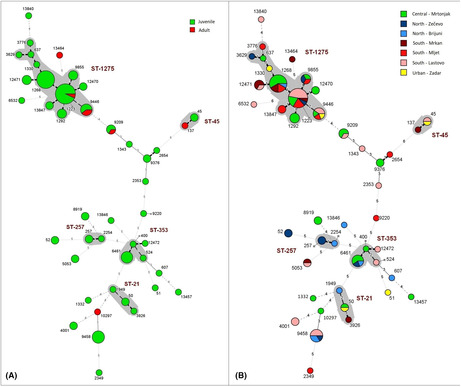

## INTRODUCTION

Birds are considered natural reservoirs for *Campylobacter* spp., which occur in the microbiota of their digestive tract (Sheppard et al., 2011). The presence of *Campylobacter* spp. has been confirmed in various wild bird groups, including waders (Charadriidae, Scolopacidae), gulls (Laridae), ducks and geese (Anatidae), thrushes (Turdidae), and birds of prey (Accipitridae). This occurrence is largely influenced by feeding habits, behaviour, and migration patterns (Broman et al., 2004; Colles et al., 2009; Griekspoor et al., 2013; Hald et al., 2016; Hock et al., 2023; Waldenström et al., 2002, 2010). Studies indicate that opportunistic bird species and those frequently feeding on the ground show a higher prevalence of *Campylobacter* spp. (Hock et al., 2023; Waldenström et al., 2002). The yellow‐legged gull (*Larus michahellis*) is one such species, living close to humans and often utilizing anthropogenic food sources from landfills, city parks, and fishing ports (Duhem et al., 2003; Ječmenica et al., 2023; Méndez et al., 2020). This is the most common seabird species in Croatia, which traditionally bred on uninhabited islands until 2004 when nests began to appear in urban areas on rooftops (Jurišić pers. obs.). They lay an average of three eggs, with an incubation period of 28–30 days. The nestlings are precocial and semi‐nidifugal, typically leaving the nest after 3 days but remaining in the nesting area (Cramp & Simmons, 1980).

There is limited information on how bacterial communities in the gut of wild birds change with age. Birds are colonized by bacteria shortly after hatching, and microbial populations shift as nestlings age, influenced by bacteria type (Awad et al., 2016; Mills et al., 1999). Nestlings ingest microbes from regurgitated food, adult saliva, and the nest environment, with the microbial population also changing through mutual competition among bacteria (González‐Braojos et al., 2012; Indykiewicz et al., 2021). In poultry, the prevalence of *Campylobacter* is a significant concern, as poultry meat consumption is a primary source of campylobacteriosis in humans (EFSA and ECDC, 2022). Studies on chickens (*Gallus gallus*) show that they are most susceptible to *Campylobacter* colonization at 2–3 weeks of age, though some may be colonized shortly after hatching. There is a small percentage of chickens that remain persistent shedders. In addition, some never become infected with *Campylobacter* spp. (Achen et al., 1998; Bull et al., 2006; Colles et al., 2011).

This study aims to determine the occurrence and genetic diversity of three *Campylobacter* species (*Campylobacter jejuni*, *Campylobacter lari*, and *Campylobacter coli*) isolated from yellow‐legged gulls in different parts of Croatia and to explore differences between adults and nestlings. We hypothesize that nestlings will have a similar prevalence and genetic diversity as adults due to their feeding methods and aim to investigate differences between sampling sites.

## EXPERIMENTAL PROCEDURES

### Catching gulls and sampling

**FIGURE 1:**
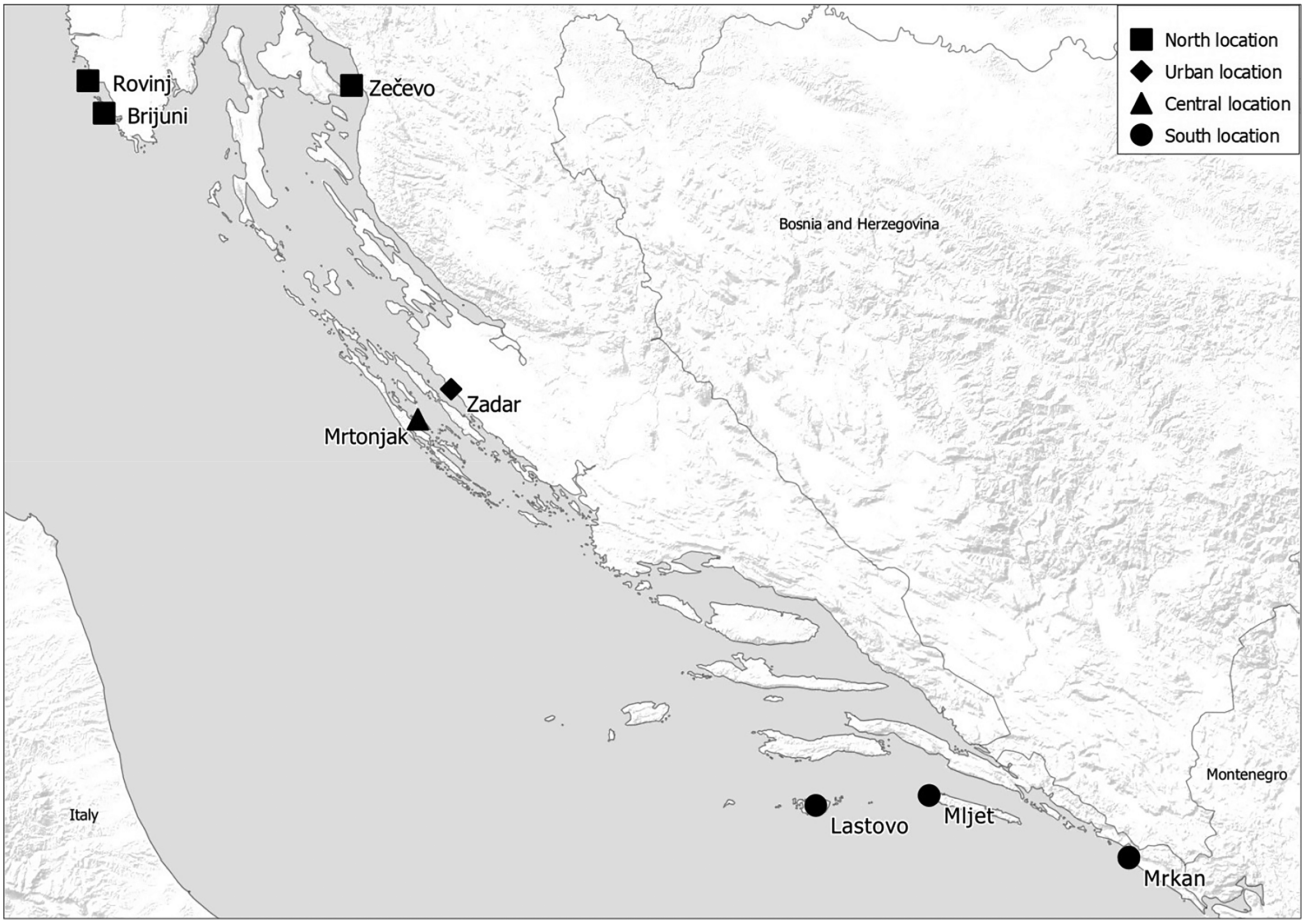
Sampling sites of yellow‐legged gulls, *Larus michahellis* in Croatia. Sites are divided into four areas according to geographical distribution and ecology to north, urban, central, and south. Map was made using QGIS.org, 2021.

From 2021 to 2023, gulls were captured in eight breeding colonies (Figure 1). Adults were captured using ‘walk‐in’ traps at active nests with incubating eggs, while nestlings were caught before fledging by hand. Each bird was ringed with metal and plastic colour rings, and cloacal swabs were taken using Amies swabs with charcoal (Copan). Samples were stored at 4°C and cultured within 3 days. Permission for gull trapping and sampling was granted by the Ministry of Economy and Sustainable Development of Croatia (UP/I‐612‐07/21‐48/99, UP/I‐352‐04/23‐08/111).

Sampling sites were categorized into three geographical groups within the Adriatic: north, central, and south. All colonies were located on natural, uninhabited islands, except for one urban colony in Zadar (Figure 1).

### *Isolation and identification of* Campylobacter *spp.*

Isolation followed the EN ISO 10272‐1:2017 method (Anonymous, 2017). Swabs were inoculated onto selective media modified charcoal cefoperazone deoxycholate, oxoid and CampyFood agar, (bioMerieux), and incubated in a microaerophilic environment at 41.5°C for 44 ± 4 h. Characteristic colonies were then plated on nutrient Columbia blood agar (bioMerieux, 10% sheep blood) and grown under the same conditions at 41.5°C for 24–48 h. Species identification was performed by PCR, targeting the *hipO* gene of *C. jejuni*, *gly*A gene of *C. coli*, *cpn*60 gene of *C. lari*, and 23S rRNA gene of *Campylobacterales* (Ferrari et al., 2023).

DNA was extracted from colonies stirred in 100 μL Dnase/Rnase‐free water, heated to 95°C for 20 min, and centrifuged at 14,000 rpm for 1 min. DNA amplification was performed using Alpha Cycler 1 (PCRmax UK) under the conditions specified by Ferrari et al. (2023). PCR results were visualized via electrophoresis on a 2% agarose gel.

### *Sequencing and genotyping of* Campylobacter *spp.*

The multilocus sequence typing (MLST) method was employed to genotype isolated *Campylobacter* bacteria. This involved sequencing seven housekeeping genes for *C. jejuni* and *C. coli*, and a different set for *C. lari* (Dingle et al., 2001; Miller et al., 2005). Sequenced DNA fragments were processed by Macrogen Europe (Netherlands). For samples where MLST failed, whole‐genome sequencing was conducted using the NucleoSpin Microbial DNA Kit and sequenced at MicrobesNG (Birmingham, UK) via Illumina short‐read sequencing.

Allele numbers were assigned based on the pubMLST database for *C. jejuni*/*coli* and *C*. non‐*jejuni*/*coli* (Jolley et al., 2018), resulting in a seven‐digit numerical code known as sequence type (ST). Each ST was categorized into a clonal complex (CC) if present in the pubMLST database. CC stands for a group of two or more independent STs that have identical alleles at least four or more gene loci (Colles & Maiden, 2012).

Minimum spanning trees for categorical data were generated with BioNumerics 8.1 (bioMérieux, Applied Maths, Belgium) using advanced cluster analysis on trait data with bootstrap resampling of 1000 samples and high‐score summary method.

### Statistical analyses

Birds were divided into nestlings and adults (breeding birds at least 4 years old). Analyses were conducted using R software 4.2.1 (R Core Team, 2022).

Due to the small number of positive cases and the large differences in sample sizes, Firth's logistic regression (bias‐reduced penalized‐likelihood) was applied using the logistf package (Heinze et al., 2023). This method minimizes the bias caused by small samples, rare events, and complete separation (Heinze & Puhr, 2010; Heinze & Schemper, 2002). The response variable was the ratio between occurrence and non‐occurrence of *Campylobacter* bacteria (1 = positive sample; 0 = negative sample). Likelihood ratio test values and p‐values summarized the results. Models were compared using anova.logistf (Heinze et al., 2023).

The Simpson's diversity index (1‐D) was used with the vegan package (Oksanen et al., 2022) to compare the diversity of *C. jejuni* and *C. lari* according to age and location. The value of the index ranges from 0 to 1, with higher values indicating greater diversity.

The similarity of genotypes by location was assessed using the proportional similarity index (PSI) with the epiR package (Stevenson & Sergeant, 2023). The PSI also ranges from 0 to 1, where 1 indicates that the distribution of types between the two matrices is identical, and 0 indicates no similarity (Garrett et al., 2007).

Due to the small number of *C. coli* isolates (*N* = 10), no statistical analysis was performed.

## RESULTS

### Occurrence

Out of 1071 gulls sampled, 152 bacterial isolates were identified as *Campylobacter* spp. (14.19%). *C. jejuni* was the most frequent (9.90%), followed by *C. lari* (3.36%) and *C. coli* (0.93%) (Table 1). Among adults (*N* = 191), 3.66% were infected with *C. jejuni* and 9.42% with *C. lari*, while no *C. coli* was found. Among nestlings (*N* = 880), 11.25% were infected with *C. jejuni*, 2.05% with *C. lari*, and 1.14% with *C. coli*.

**TABLE 1:** The number of yellow‐legged gulls sampled and the number of positive *Campylobacter* spp. samples across eight breeding colonies in Croatia, differentiated by age and species of *Campylobacter*.

	Location	Rovinj	Brijuni	Zecevo	Zadar	Mrtonjak	Lastovo	Mljet	Mrkan	Total
	No. samples/age	46	84	134	57	178	191	174	207	1071
*C. jejuni*	Adult	0	0	0	1	3	1	0	2	7
	Nestlings	0	8	10	4	32	23	13	9	99
*C. lari*	Adult	3	0	5	0	4	4	0	2	18
	Nestlings	0	2	1	0	10	3	1	1	18
*C. coli*	Adults	0	0	0	0	0	0	0	0	0
	Nestlings	0	2	2	0	4	0	0	2	10
No. samples	Adult	8	24	30	18	35	30	7	39	191
	Nestlings	38	60	104	39	143	161	167	168	880
*C. jejuni* %		0.00	9.52	7.46	8.77	19.66	12.57	7.47	5.31	9.90
*C. lari* %		6.52	2.38	4.48	0.00	7.87	3.66	0.57	1.45	3.36
*C. coli* %		0.00	2.38	1.49	0.00	2.25	0.00	0.00	0.97	0.93
*Campylobacter* spp. %		6.52	14.29	13.43	8.77	29.78	16.23	8.05	7.73	14.19

Mrtonjak had the highest occurrence of *Campylobacter* spp. (29.78%), followed by Lastovo (16.23%), Brijuni (14.29%), and Zečevo (13.43%). Other locations had less than 10% occurrence. *C. jejuni* and *C. lari* were present at all sites, while *C. coli* was found at four sites (Table 1).

Firth's logistic regression revealed a significant difference in *Campylobacter* occurrence depending on the sampling site, while age was significant in all models except for overall positive samples (Table 2). Significant differences were noted for the occurrence of *Campylobacter* spp. (*X* ^2^ = 46.2, *df* = 6, *p* <0.001) and *C. jejuni* (*X* ^2^ = 19.11, *df* = 6, *p* = 0.004). No significant difference was found between the models for the occurrence of *C. lari*, which means that the data are not sufficient to make a statement about how location and age influence the occurrence of *C. lari* in yellow‐legged gulls.

**TABLE 2:** Association between age and colonies of yellow‐legged gulls sampled and the occurrence of *Campylobacter* spp., *C. jejuni*, and *C. lari* using Firth's bias‐reduced penalized‐likelihood logistic regression.

Response	Predictor	*X* ^2^	Df	*p*‐value
*Campylobacter* spp. (*n* = 1071)	Age	0.19	1	0.67
	Location	46.48	7	< 0.001*
*C. jejuni* (*n* = 1071)	Age	11.65	1	< 0.001*
	Location	30.77	7	< 0.001*
*C. lari* (*n* = 1071)	Age	20.61	1	< 0.001*
	Location	19.94	7	0.006*

*Note*: *indicates significant difference (*p* <0.05).

### Genotypes

MLST was performed on all 152 isolates, generating complete ST profiles for 141 isolates. Of these, 6 *C. jejuni*, 10 *C. lari*, and 8 *C. coli* yielded complete ST profiles after whole genome sequencing. However, some genomes could not be closed due to missing loci, preventing correct ST assignment. The de novo assemblies consisted of varying numbers of contigs, resulting in incomplete chromosome sequences for some isolates. Missing even a single locus prevented the assignment of the correct ST.

Out of 106 *C. jejuni* isolates, 100 yielded complete allelic profiles, belonging to 44 known STs, while 11 new STs were identified. Seventy‐two STs were grouped into 14 different CCs, leaving 28 as singletons. The most common CC found in *C. jejuni* was ST‐1275 (*N* = 43; 43%). For *C. lari*, 33 of 36 isolates had complete profiles, with five new STs. They belonged to 20 different STs, none assigned to a CC. Of ten *C. coli* isolates, eight had complete profiles, belonging to five known STs and one new ST. Five STs belonged to two CCs, while one new ST was unassigned. The most frequent CC was ST‐828 (*N* = 6; 75%).

Simpson's diversity index showed high diversity (>0.90) for *C. jejuni* at all sites except the urban area (*N* = 5, 1‐D = 0.80) (Table 3). *C. lari* showed slightly lower diversity, influenced by fewer isolates (Table 4). Both *C. jejuni* and *C. lari* had highest diversity in the south area (Tables 3 and 4).

**TABLE 3:** Simpson's indexes (1‐D) and proportional similarity indexes (PSI) for *C. jejuni* genotypes isolated from yellow‐legged gulls in relation to the location of breeding colonies.

Area	1‐D	Location	PSI						
			Brijuni	Zečevo	Mrtonjak	Zadar	Lastovo	Mljet	Mrkan
North	0.90	Brijuni	1						
		Zečevo	0.22	1					
Central	0.90	Mrtonjak	0.22	0.22	1				
Urban	0.80	Zadar	0	0	0.06	1			
South	0.91	Lastovo	0.13	0.22	0.17	0.13	1		
		Mljet	0.13	0.19	0.31	0.08	0.31	1	
		Mrkan	0.13	0.11	0.31	0	0.19	0.26	1

**TABLE 4:** Simpson's indexes (1‐D) and proportional similarity indexes (PSI) for *C. lari* genotypes isolated from yellow‐legged gulls in relation to the location of breeding colonies.

Area	1‐D	Location	PSI						
			Rovinj	Brijuni	Zečevo	Mrtonjak	Lastovo	Mljet	Mrkan
North	0.86	Rovinj	1						
		Brijuni	0	1					
		Zečevo	0.2	0.2	1				
Central	0.85	Mrtonjak	0.08	0.17	0.45	1			
South	0.91	Lastovo	0.14	0	0	0.08	1		
		Mljet	0	0	0	0	0	1	
		Mrkan	0	0	0.40	0.25	0	0	1

PSI analysis showed low overall similarity between sites, with the highest PSI of 0.31 and 0.45 for *C. jejuni* and *C. lari*, respectively. *C. jejuni* showed higher PSI between south and central sites, while for *C. lari* central site (Mrtonjak) was similar to north (Zečevo) and south (Mrkan) locations (Tables 3 and 4).

**FIGURE 2:**
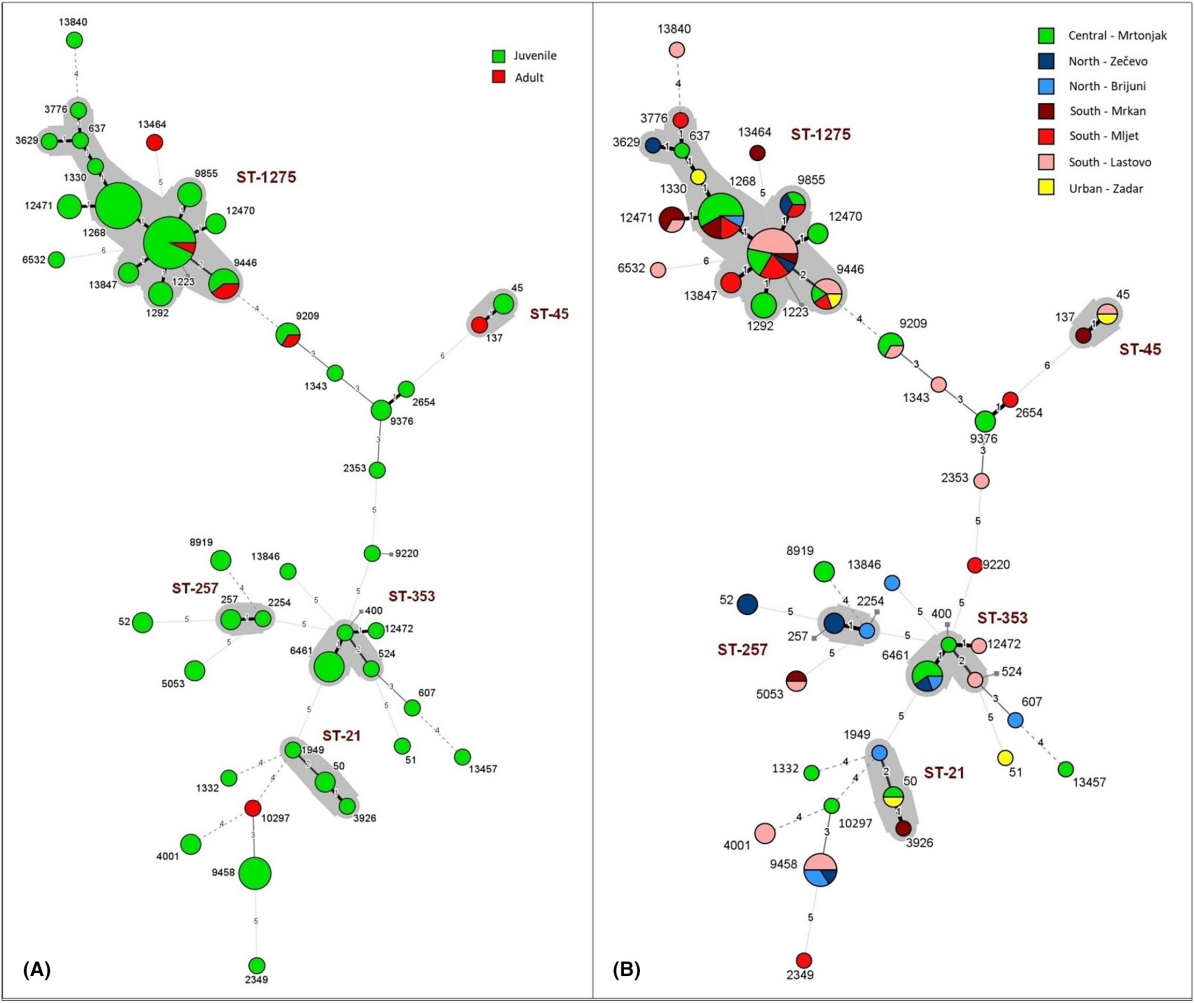
Minimum spanning tree with identified *C. jejuni* STs by age group (A) and locations (B). Isolates are labelled by sequence type and most frequent clonal complex (grey). Differences between samples are expressed as the number of allele differences between them (number of locus variants). The relationship is visualized as follows: a thicker solid line corresponds to 1 and 2 locus variants; a thinner solid line corresponds to 3 locus variants; a thinner dashed line corresponds to 4 locus variants and a thinner dotted line corresponds to 5 and 6 locus variants.

**FIGURE 3:**
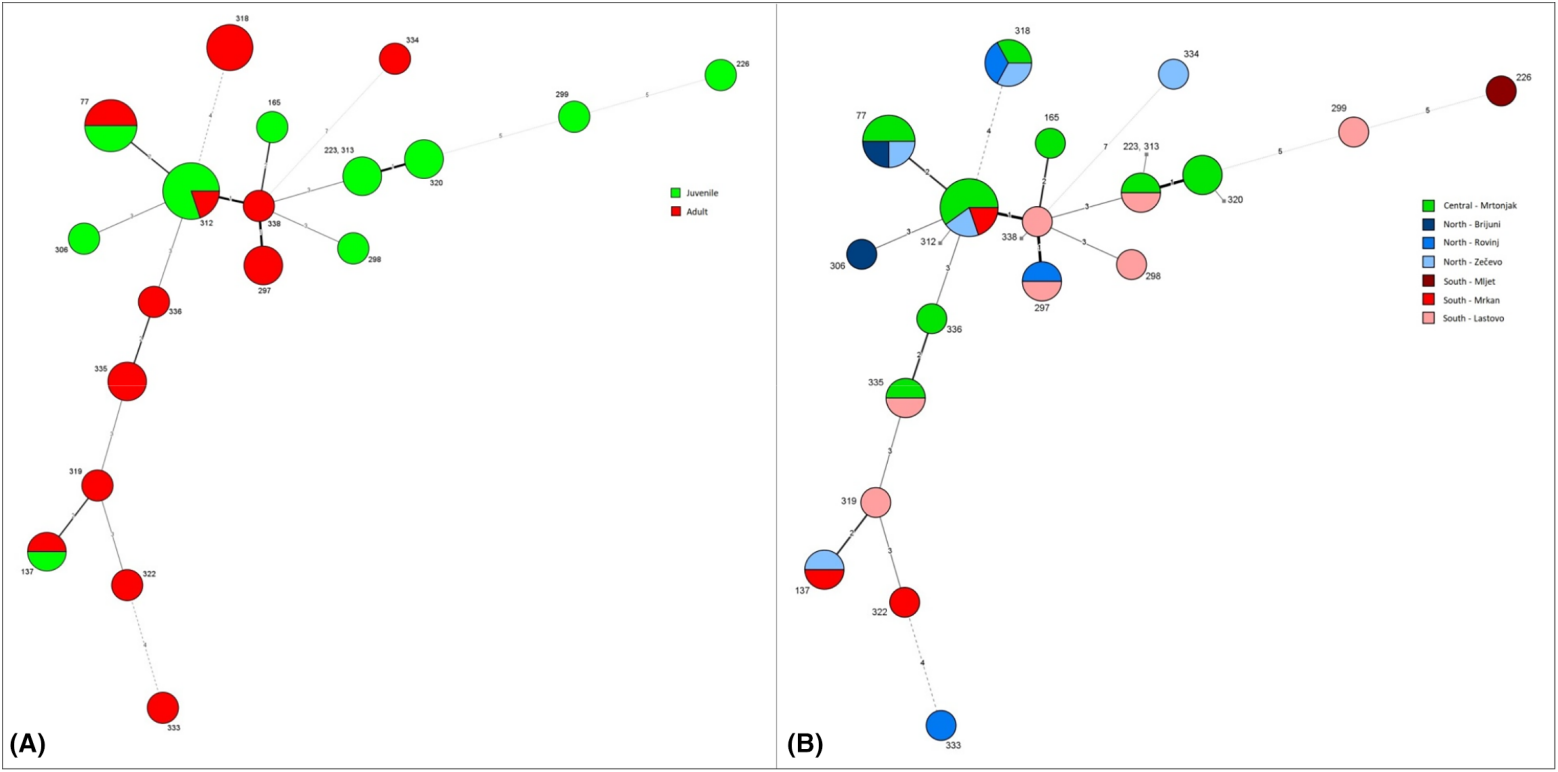
Minimum spanning tree with identified *C. lari* STs by age group (A) and locations (B). Isolates are labelled by sequence type. Differences between samples are expressed as the number of allele differences between them (number of locus variants). The relationship is visualized as follows: a thicker solid line corresponds to 1 and 2 locus variants; a thinner solid line corresponds to 3 locus variants; a thinner dashed line corresponds to 4 locus variants and a thinner dotted line corresponds to 5 and 7 locus variants.

Minimum spanning trees, showed no significant genotype clustering by age or sites for both *C. jejuni* or *C. lari* (Figures 2 and 3).

Both adult and nestlings had high Simpson's diversity indexes for *C. jejuni* and *C. lari*. (adult 0.82 and nestlings 0.94 for *C. jejuni*; adult 0.90 and nestlings 0.88 for *C. lari*). Three STs (1223, 9209, 9446) were found in both adults and nestlings for *C. jejuni*, and three STs (77, 137, 312) for *C. lari* (Figures 2 and 3). For *C. jejuni*, ST 1223 was consistently found in Lastovo, Mrtonjak, and Mljet across all three survey years, and in Zečevo in 2022. ST 9446 appeared each year but in different gull colonies, while ST 9209 was found in Mrtonjak in 2022 and 2023, and also in Lastovo in 2023. For *C. lari*, ST 77 was identified in Mrtonjak in 2021 and 2022, and in Zečevo and Brijuni in 2023. ST 137 was only found in Mrtonjak in 2023. ST 312 was present in Mrtonjak and Zečevo in 2021 and 2023, and in Mrkan in 2022.

## DISCUSSION

Our results show that the total occurrence of *Campylobacter* spp. in yellow‐legged gulls was 14.19%, with *C. jejuni* being the most frequently detected species, followed by *C. lari* and *C. coli*, which were very rarely detected and only in nestlings. The occurrence of *Campylobacter* in yellow‐legged gulls varies greatly across studies. For example, Antilles et al. (2021) reported a very low incidence of *Campylobacter* spp. at around 1%, whereas Migura‐Garcia et al. (2017) found an occurrence of 10.4%, with *C. jejuni* being the most frequently detected species in both studies. In contrast, Russo et al. (2021) reported a higher incidence of *Campylobacter* spp. at 26.7%, with *C. coli* being the most frequently isolated species, followed by *C. jejuni*. Our findings are comparable to those for other gull species (Jurinović et al., 2020; Keller & Shriver, 2014; Moore et al., 2002; Moré et al., 2017). However, there are exceptions, such as the black‐headed gull (*Larus ridibundus*), which exhibited a higher incidence of 36.2%, and the Audouin's gull (*Larus audouinii*), with occurrences ranging between 2% and 31.8% (Antilles et al., 2021; Broman et al., 2002).

*C. jejuni* is the most frequently reported species of the entire genus causing campylobacteriosis in humans, which is the most frequently reported zoonosis in the European Union (EFSA and ECDC, 2022). It is commonly found in wild birds, which is confirmed with our results, but it is also widespread in humans, poultry, other farm animals, and in the environment (Colles et al., 2003, 2009; Dingle et al., 2001, 2005; Hermans et al., 2011; Keller & Shriver, 2014; Lévesque et al., 2008; Mulder et al., 2020).

*C. lari* is frequently isolated from gulls, other seabirds, and shellfish (Lozano‐León et al., 2021; Matsuda & Moore, 2011; Moore et al., 2002; Rincé et al., 2018; Waldenström et al., 2006). *C. lari* ability to survive longer in seawater may explain its relatively high abundance in yellow‐legged gulls (Obiri‐Danso et al., 2001).

The low occurrence of *C. coli* suggests that it does not frequently colonize yellow‐legged gulls, other gull species, or wild birds in general (Broman et al., 2002; Colles et al., 2009; Hald et al., 2016; Hughes et al., 2009; Jurinović et al., 2020; Waldenström et al., 2002). Like *C. jejuni*, *C. coli* is widespread in humans, animal husbandry, and the environment (Andrzejewska et al., 2022; Dingle et al., 2005; Mulder et al., 2020).

High genetic diversity of *Campylobacter* spp. was found in yellow‐legged gulls, comparable to other gull species and wild birds (Colles et al., 2011; Hughes et al., 2009; Keller & Shriver, 2014).

Some genotypes may adapt to specific hosts or environments (Dingle et al., 2005; Gourmelon et al., 2022; Griekspoor et al., 2013; Hepworth et al., 2011; Hughes et al., 2009). However, genetic studies show that wild birds harbour strains similar to those found in humans, livestock, and the environment (Gourmelon et al., 2022; Griekspoor et al., 2013; Wei et al., 2019). Our results suggest that most of the CC for *C. jejuni* found in gulls are also present in other sources. The most common CC identified in our study, ST‐1275, was frequently found in wild birds, almost exclusively in gulls, including yellow‐legged gulls. Other CCs identified, such as ST‐21 and ST‐45, are commonly isolated from human stools and chickens (Iglesias‐Torrens et al., 2018; Jolley et al., 2018; Keller & Shriver, 2014). Even when gulls share CCs with other sources, the isolates are mostly genetically distinct (Jurinović et al., 2020).

A few identified *C. coli* isolates mostly belong to ST‐828, which has been found in human stool and pigs (Dingle et al., 2005). *C. lari* is still an understudied bacterium compared to *C. jejuni* and *C. coli*, with only 855 isolates in the PubMLST database for *C*. non‐*jejuni*/*coli* (Jolley et al., 2018). Many new genotypes are being discovered, but their diversity makes it difficult to assign many CCs to this species (Jolley et al., 2018; Jurinović et al., 2023). In our study, 65% of STs were found only for the second or third time according to the database. Of the 20 STs recorded in the present study, 50% were found only in gulls from Croatia, 30% were found in gulls and other wild birds, human stool, and undefined food, and 20% were novel STs (Jolley et al., 2018; Jurinović et al., 2023). This highlights the high genetic diversity of *C. lari* which was also observed by Gourmelon et al. (2022), suggesting that new subspecies and species within *C. lari* group remain to be identified.

Regarding age‐related occurrence, given the small and uneven sample size, there were no significant differences in the number of positive samples between nestlings and adult birds. This is expected due to the feeding method, as the food is regurgitated by the parents. Similar results were found for black‐headed gulls and European starlings (*Sturnus vulgaris*) (Colles et al., 2009; Indykiewicz et al., 2021). However, there was a significant difference in the occurrence of *C. jejuni*, with nestlings showing a higher occurrence than adults, while no significant difference was found for *C. lari*. Additionally, nestlings were the only ones carrying *C. coli*.

Several factors could influence the differences in the incidence of *Campylobacter* species between adults and nestlings. Horizontal transmission between young birds from different nests is likely when they move around the colony, while adult birds have limited contact with other birds after copulation, making horizontal transmission less probable (Indykiewicz et al., 2021). Furthermore, faecal matter accumulates in the colony over time, increasing the amount of bacteria to which the nestlings are exposed (Benskin et al., 2009). As the nestlings grow older, adult birds spend less time in the colony, reducing their exposure to bacteria.

The lower immunity of nestlings could be another reason for the higher occurrence of *Campylobacter* and the presence of different bacterial species in nestlings compared to adults. The study by Han et al. (2016) on poultry, suggests that the colonization rate of *C. jejuni* is influenced by the age of the chickens and the maturation of their immune system, as well as to some extent by the developing gut microbiota. They also found differences in the colonization and immune response of different *C. jejuni* strains, highlighting the complexity of the relationship between bacteria and host. Additionally, Cawthraw and Newell (2009) suggest that maternal antibodies play an important role in protecting against *Campylobacter* colonization in the early life stages of chickens.

Further studies are needed to understand how the occurrence of *Campylobacter* and other bacteria changes with the age of birds and whether they carry the same genotypes over time. Given the high genetic diversity of our samples, there appears to be a rapid turnover of genotypes, as noted by other researchers (Broman et al., 2002; Colles et al., 2009). However, we found that a small number of genotypes were consistently present in this gull population throughout the study years, such as ST‐1275 in *C. jejuni*, suggesting longevity and host specificity of certain genotypes.

Significant differences in the occurrence of *Campylobacter* were found between the sites. The island of Mrtonjak in the central Adriatic had the highest occurrence, though the reason for this is unknown. Most *C. jejuni* and *C. coli* isolates belong to the CCs found in humans, suggesting that gulls may be more exposed to these pathogenic bacteria due to their frequent feeding in anthropogenic habitats. This exposure potentially contributes to the spread of these bacteria to other habitats and animals (Gourmelon et al., 2022; Griekspoor et al., 2013; Hughes et al., 2009; Jurinović et al., 2020).

However, considering that yellow‐legged gulls are a highly mobile species utilizing various habitats and that all studied colonies are close to human settlements, the risk of *Campylobacter* infection should be equally high for all colonies (Ječmenica et al., 2023; Kralj et al., 2013). Indykiewicz et al. (2021) found no significant differences in the occurrence of *Campylobacter* between urban and rural black‐headed gull colonies, as both areas are exposed to the pathogen. In our study, we could not properly compare urban areas with natural areas due to the small number of samples collected. Nevertheless, we support the hypothesis that most genotypes of *C. jejuni* and *C. coli* belong to isolates found in humans and chicken meat, indicating that both urban and natural habitats are exposed to *Campylobacter* spp.

In terms of genetic diversity, the minimum spanning trees show no significant differences between age groups and locations. There is no clustering of specific genotypes by area or age groups, suggesting that all sampled yellow‐legged gulls have similar exposure to certain *Campylobacter* genotypes.

Although both adults and nestlings had a high Simpson's diversity index, nestlings had a higher diversity of *C. jejuni* than adults (0.94 and 0.82, respectively). In a study of free‐range poultry, it was suggested that birds are exposed to an increasing number of *Campylobacter* genotypes that accumulate in the flock and breeding colonies, which could explain the slightly higher diversity observed in nestlings (Colles et al., 2011).

The PSI shows varying degrees of similarity of isolates according to their allelic profiles between sites in all areas. For both *C. jejuni* and *C. lari*, there is stronger similarity between different sites, suggesting a regular *Campylobacter* flow between different parts of Croatia. Overall, we could not find a genotype‐specific to the age or breeding location of the birds.

In conclusion, considering the low number of positive samples and the smaller number of adult birds compared to nestlings, the analysis showed that nestlings had a significantly higher occurrence of *C. jejuni*. In addition, significant differences in the occurrence of *Campylobacter* spp. were found between the individual breeding sites.

There is a high genetic diversity of *C. jejuni* and *C. lari* with different and previously undescribed genotypes, which emphasizes how little research has been done on *Campylobacter* spp. in wild birds. Similar to other studies, our results suggest that *C. jejuni* and *C. lari* have high genetic diversity, with genotypes specific to wild birds. However, potentially pathogenic strains can also be found (Wei et al., 2019).

Birds are considered one of the most important sources of *Campylobacter*, and there are documented cases where birds have contaminated and transmitted these bacteria to the environment and other animals (Mulder et al., 2020). Although these cases are rare, it is important for public health to understand the role of wild birds in the epidemiology of *Campylobacter* and there is a need for future studies.

## AUTHOR CONTRIBUTIONS

**Biljana Ječmenica:** Conceptualization; investigation; writing – original draft; formal analysis; visualization; methodology. **Sanja Duvnjak:** Formal analysis; writing – review and editing; visualization. **Andrea Humski:** Writing – review and editing. **Louie Thomas Taylor:** Writing – review and editing; methodology; investigation. **Jelena Kralj:** Writing – review and editing; formal analysis; investigation. **Fani Krstulović:** Writing – review and editing. **Tajana Amšel Zelenika:** Writing – review and editing. **Viktor Mašović:** Writing – review and editing. **Luka Jurinović:** Conceptualization; investigation; writing – review and editing; supervision; methodology; funding acquisition.

## CONFLICT OF INTEREST STATEMENT

The authors declare no conflict of interest.

## Data Availability

The data that support the findings of this study are available on request from the corresponding author.
